# In-depth investigation of the molecular pathogenesis of bladder cancer in a unique 26-year old patient with extensive multifocal disease: a case report

**DOI:** 10.1186/1471-2490-10-5

**Published:** 2010-02-26

**Authors:** Tahlita CM Zuiverloon, Cheno S Abas, Kirstin A van der Keur, Marcel Vermeij, Stephen S Tjin, Angela G van Tilborg, Martijn Busstra, Ellen C Zwarthoff

**Affiliations:** 1 Department of Pathology, Erasmus MC, Rotterdam, the Netherlands; 2 Department of Urology, Erasmus MC, Rotterdam, the Netherlands

## Abstract

**Background:**

The molecular characteristics and the clinical disease course of bladder cancer (BC) in young patients remain largely unresolved. All patients are monitored according to an intensive surveillance protocol and we aim to gain more insight into the molecular pathways of bladder tumors in young patients that could ultimately contribute to patient stratification, improve patient quality of life and reduce associated costs. We also determined whether a biomarker-based surveillance could be feasible.

**Case Presentation:**

We report a unique case of a 26-year-old Caucasian male with recurrent non-muscle invasive bladder tumors occurring at a high frequency and analyzed multiple tumors (maximal pTaG2) and urine samples of this patient. Analysis included *FGFR3 *mutation detection, FGFR3 and TP53 immunohistochemistry, mircosatellite analysis of markers on chromosomes 8, 9, 10, 11 and 17 and a genome wide single nucleotide polymorphism-array (SNP). All analyzed tumors contained a mutation in *FGFR3 *and were associated with FGFR3 overexpression. None of the tumors showed overexpression of TP53. We found a deletion on chromosome 9 in the primary tumor and this was confirmed by the SNP-array that showed regions of loss on chromosome 9. Detection of all recurrences was possible by urinary *FGFR3 *mutation analysis.

**Conclusions:**

Our findings would suggest that the BC disease course is determined by not only a patient's age, but also by the molecular characteristics of a tumor. This young patient contained typical genetic changes found in tumors of older patients and implies a clinical disease course comparable to older patients. We demonstrate that *FGFR3 *mutation analysis on voided urine is a simple non-invasive method and could serve as a feasible follow-up approach for this young patient presenting with an *FGFR3 *mutant tumor.

## Background

Bladder cancer (BC) is a disease of the elderly with a peak incidence in the sixth decade of life. Tumors are sporadically found under the age of 40 (1-4%) and most young patients present with tumors of low stage and grade [1-4]. Conflicting results have been found concerning the natural history and prognosis of bladder tumors in young patients. The small number of cases and the definition of "young" with age ranging from 5-45 years may be responsible for this variation [2,3,5,6]. Evidence is accumulating that there is a difference in the natural history of patients under the age of 20 and patients between 30-50 years of age. Patients <20 years mostly have tumors with a low recurrence rate, a favorable clinical outcome and few genetic alterations, while patients between 30-50 years have a disease course comparable to older patients [7].

Almost 80% of the BC patients will present with non-muscle invasive disease (NMI-BC). Treatment is by trans-urethral resection of the tumor, but almost 70% of the patients will have at least one recurrence within five years and 10-20% will progress to muscle-invasive disease. After the first tumor resection all age groups of patients are monitored according to an intensive surveillance protocol that includes 3-monthly cystoscopies the first two years, followed by less frequent observations if a patient stays recurrence free. The main disadvantages of the current protocol are life-long invasive and costly cystoscopic monitoring of patients causing physical discomfort and sexual dysfunction [8].

To our knowledge there are only few studies that investigated the molecular changes in bladder tumors of young patients. Identifying the molecular pathways of these tumors could define a subset of patients and redirect patient management towards a new patient friendly and individualized follow-up protocol. One of the most promising markers associated with NMI bladder tumors is the mutation status of the Fibroblast Growth Factor Receptor-3 (*FGFR3*). Mutations in *FGFR3 *have been associated with BC tumors of low stage and grade and patients having a favorable prognosis [9]. We have recently shown that *FGFR3 *mutation analysis on voided urine of NMI-BC patients with a mutation in *FGFR3 *is a non-invasive inexpensive tool for patient surveillance (Zuiverloon *et al*. submitted). Additionally, multiple studies report on the use of microsatellite analysis (MA) for detection of loss-of-heterozygosity (LOH) as a diagnostic marker. LOH detected by MA is mainly located on chromosomes 8, 9, 10, 11, 13 and 17 and these losses have been associated with stage, grade, invasive growth, recurrent disease and progression. In the present study we analyzed multiple tumor and urine samples of a unique young patient for *FGFR3 *mutation status, LOH, FGFR3 and TP53 expression and performed a genome wide single nucleotide polymorphism-array (SNP). Since this patient presented with multiple recurrences within a short time-span we determined retrospectively whether *FGFR3 *mutation detection could be a feasible follow-up approach.

## Case presentation

We report a unique case of a 26-year-old Caucasian male with recurrent non-muscle invasive bladder tumors occurring at a high frequency. The patient presented at first in March 2007 with macroscopic hematuria for a few weeks. He received a total of 5 trans-urethral resections of the multifocal bladder tumors within 2 years, highest stage and grade being TaG2. The first three resections included an average of 15 papillary tumors and the last two resections included 3 tumors. The patient received two direct post-operative intravesical instillations of epirubicin. Additionally, our patient initially received adjuvant intravesical immunotherapy with bacillus calmette-guérin (BCG) in 2008, but switched to mitomycin-C (MMC) in 2009 for maintenance due to complications. A CT-scan of the pelvis and abdomen demonstrated no evidence of upper urinary tract lesions, no signs of urolithiasis, nodal or distant metastases. There was no family history of bladder cancer and intoxications included a smoking status of 5.5 pack-years (one pack-year = one pack of cigarettes a day for one year ~20 cigarettes a day for one year). There was no indication of any contact with aromatic amines. The patient worked as a soldier in Bosnia in 2001 and 2003 where he was part of a recovery team driving a diesel armed-truck. Tasks of the recovery team included cleaning of remaining ammunition enforced with depleted uranium (DU) and military equipment wreckage.

## Methods

### Tissue samples

Tumor tissue was obtained from formalin-fixed, paraffin-embedded samples. Tumor sections were selected by pathological examination to contain a minimum amount of 80% tumor cells and sections were manually dissected from 4 μ slides. Samples were first deparaffinized and DNA was extracted using the Qiagen Dneasy blood and tissue kit (Qiagen, GmbH, Hilden, Germany) according to the manufacturer's protocol.

### Urine samples

Freshly voided urine (10-100 ml) was collected prior to a cystoscopy or trans-urethral resection of the tumor and stored at 4°C until transportation to the department of Pathology at Erasmus MC, Rotterdam. Urine was spun down for 10' at 3000 rpm (1500 × g). Cell pellets were washed twice with 10 ml of Phosphate-buffered saline (PBS) and spun down for 10' at 3000 rpm. Pellets were resuspended in 1 ml of PBS, transferred to an eppendorf vial and spun down for 5' at 6000 rpm (3000 × g). Supernatant was discarded and the cell pellet was stored at -20°C until DNA isolation. DNA was extracted using the QiAamp DNA mini-kit (Qiagen GmbH, Hilden, Germany) according to the manufacturer's protocol.

### Fibroblast growth factor receptor 3 mutation analysis

The *FGFR3 *mutation detection assay was performed as described previously by van Oers *et al*. [10]. In short a multiplex PCR of the three regions that contain the most frequent FGFR3 mutations (exon 7, 10 and 15) was performed. This was followed by a single nucleotide polymorphism analysis using probes that anneal to the PCR product adjacent to the mutation site. Probes were extended with a labeled dideoxynucleotide and the products were analyzed on an automatic sequencer (ABI PRISM 3130 XL Genetic Analyzer, Applied Biosystems) with the label indicating the presence or absence of a mutation. Genescan Analysis Software version 3.7 (Applied Biosystems) was used for analysis of the data.

### Microsatellite analysis

Microsatellite analysis was performed as described by van der Aa *et al*. [11]. Markers used for detection of LOH were: D8S1130, D81125, D8S1107, D8S1109, D8S1145, D9S1118, D9S252, D9S304, D9S299, D9S752, D9S930, G10693, D10S1225, D11S1999, D11S1981 and D17S969.

### FGFR3 and TP53 immunohistochemistry

Bladder tumors were fixed in 10% buffered formaldehyde, embedded in paraffin, 4 micrometer sections were mounted on a coated glass slide (Starfrost Knittel-Glaeser D38114 Braunschweig Germany). Haematoxylin-Eosin was used as a general stain and immunohistochemistry was performed with rabbit polyclonal antibody anti-human FGFR3 (Santa Cruz Biotechnology INC) and monoclonal mouse anti-human P53 protein clone D-07 (Dakocytomation, Denmark A/S). Antigen retrieval by microwave heating in TRIS-EDTA buffer pH 9.0 was used and endogenous peroxidase was removed by 0.30% H_2_O_2 _in PBS/TWEEN. Pretreatment with blocking buffer PROTIFAR 0.5% in PBS/TWEEN was used prior to the overnight incubation at 4°C with the primary antibody. Visualization was done by Dako Real Envision Detection system peroxidase/DAB+ (Dakocytomation, Denmark A/S), containing anti-mouse and anti-rabbit antibodies, according to the manufacturer's protocol. Counterstaining was done with haematoxylin (Klinipath 6921 GX, Duiven, The Netherlands). Expression of FGFR3 was scored in a semi-quantitaive scoring system: 0 = all tumor cells negative, 1 = faint positivity of in some or all cells, 2 = weak but extensive positivity and 3 = strong positivity/overexpression (regardless of extent). TP53 overexression was scored if >10% of the cells stained positive. As a reference normal urothelium was analyzed for FGFR3 and TP53 protein expression. Reference sections of known high and low expressions levels of FGFR3 and TP53 were included in the staining runs.

### Infinium HumanHap370CNV Genome wide SNP array

For Infinium HumanHap370CNV Genotyping BeadChip SNP array analysis we used 750 ng of patient DNA and followed the protocol as described by the manufacturer (Illumina Inc., San Diego, CA, USA). We used Illumina BeadStudio software to extract data. The Nexus CGH Plug-in for CNV Analysis from BioDiscovery (BioDiscovery Inc., El Segundo, CA, USA) was used to export Illumina CNV data to Nexus Copy Number version 4.0. Arrays were processed using the built-in Rank Segmentation algorithm.

## Results and discussion

Young patients rarely present with BC and there are multiple studies that indicate a good clinical disease course where patients present with solitary tumors and a low recurrence and progression rate. Since BC patients are monitored cystoscopically according to an intensive surveillance protocol, gaining more insight into the molecular pathways of BC tumors in young patients could define a subset of patients that can be monitored less frequently, hereby improving patient quality of life and reducing associated costs.

**Figure 1:**
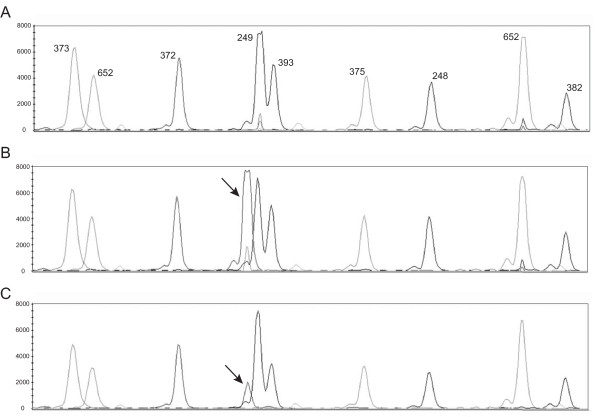
***FGFR3 *mutation detection on urinary derived DNA**. *FGFR3 *mutation analysis of an *FGFR3 *tumor without a mutation (A), mutation S249C on tumor DNA (B) and urinary derived DNA (C) from the same patient used in panel B.

**Figure 2:**
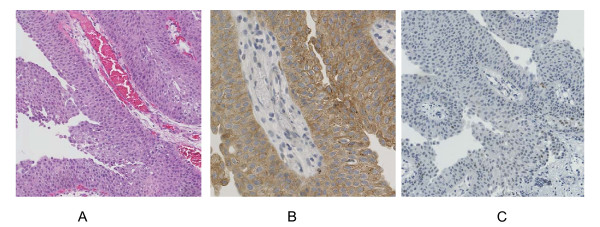
**Protein expression levels of FGFR3 and TP53 in bladder tumor tissue**. (A) Haematoxylin-Eosin staining of a papillary tumor. Original magnification ×20. (B) High levels of FGFR3 expression with a cytoplasmic and membranous patter. Original magnification ×40. (C) Low TP53 immunostaining of tumor cells showing a nuclear pattern. Original magnification ×20.

**Figure 3:**
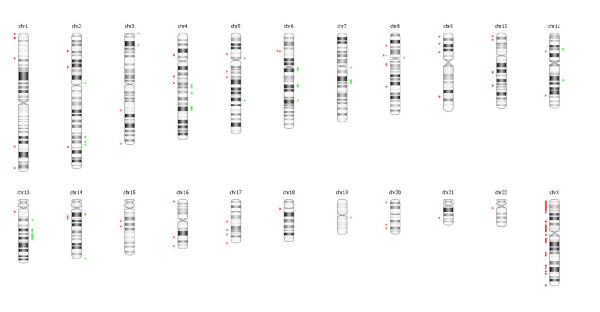
**Overview of copy number alterations for all chromosomes**. Red: loss, green: gain.

**Figure 4:**
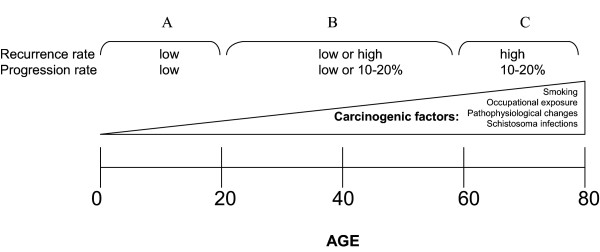
**Age related occurrence of BC due to exposure to carcinogenic factors and BC-associated genetic changes**. (A) Young BC patients, age <20, with a low carcinogenic exposure having chromosomal stable tumors, no mutations in *FGFR3 *and *TP53 *and no loss on chromosome 9. (B) Patients aged 20-60 with either a molecular profile corresponding to young (<20) or older (>60) patients. (C) Older BC patients with high exposure to carcinogenic factors and chromosomal unstable tumors, loss on chromosome 9, *FGFR3 *and *TP53 *mutations.

We presented a unique case of a 26-year-old male with multiple multifocal NMI bladder tumors recurring at a high frequency. After starting intravesical maintenance therapy with MMC the recurrence rate decreased and up to date the patient stayed recurrence free. Molecular analysis of the primary tumor revealed an S249C mutation in *FGFR3 *(Figure 1B) and overexpression of FGFR3 (Figure 2B). LOH on chromosome 9 was detected by MA and confirmed by the genome wide SNP array analysis. We also found other regions of loss and gain that are considered minor when compared to tumors of the same stage and grade (Figure 3). There was no increased expression of TP53 (Figure 2C). Hence, this young patient appears to have the typical genetic changes found in older patients with NMI-BC. This implies that the patient could have a disease course comparable to older patients and warrants regular controls due to the risk of additional recurrences or progression. These findings combined with previous studies suggest that not only a patient's age, but also the molecular characteristics of the tumor determine the clinical disease course. Since it takes time to accumulate typical genetic changes involved in BC - e.g. mutations in *FGFR3 *and *TP53 *and LOH on chromosomes 8, 9, 10, 11, 17- leading to tumor formation, most BC patients will present at an older age. Possible explanations are that older patients have a longer exposure time to BC associated exogenous risk factors and secondly that pathophysiological changes in elderly causing urinary stasis in the bladder due to urine retention lead to an increased exposure to carcinogenic substances. We suggest that this could be the reason why tumors of young BC patients mostly have few genetic alterations and may represent a biologically distinct group of tumors with an overall good clinical disease course (Figure 4A). This is in concordance with one of the few molecular studies on BC in patients <19 years (n = 14) that found no mutations in *FGFR3*, no deletions on chromosome arms 9p, 9q or 17p, no MSI and only one mutation in *TP53 *[7]. On the other hand other clinical studies of patients <40 years demonstrate a disease course comparable to older patients with typical aggressive behavior in the young presenting with a primary muscle invasive tumor, but unfortunately no molecular analyses of these tumors have been performed [2,5,12,13].

Exogenous risk factors that could have contributed to BC in our patient are smoking, exposure to diesel exhaust and depleted uranium (DU). First our patients smoking status is 5.5 pack-years, which is known to be associated with an increased risk of BC. Secondly, a meta-analysis of BC and diesel exhaust exposure demonstrated a relative risk of 1.44 for occupations exposed to high diesel fume levels [14]. Our patient worked as a driver of a diesel armed truck and was exposed to diesel exhaust fumes, working in a valley were the fumes were retained in a cloud of exploded ammunition. Lastly, although some believe that there is a link between exposure to DU and cancer development no hard evidence has been found to support this hypothesis. While evidence from Hiroshima data shows a latency period of 10-15 years to develop cancer this concerns an acute high-dose exposure and other studies were not able to demonstrate this link in Balkan veterans [15,16]. On the other hand two studies by Miller *et al*. demonstrated *in vitro *tumorigenic transformation of osteoblasts when exposed to DU [17,18].

Since Van der Aa *et al*. demonstrated that specifically young patients perceive a cystoscopic investigation as burdensome this emphasizes the need for patient stratification [19]. To determine whether a young BC patient should be monitored according to the standard follow-up protocol or can be monitored less frequently by cystoscopy, we propose to determine the *FGFR3 *mutation status of the primary tumor. One possibility is that the tumor will have few genetic changes and secondly that the tumor will have genetic changes comparable to those found in older patients (Figure 4B, 4C). Mutations in *FGFR3 *are tumor-specific and are not found in normal tissue, meaning that detection of a mutation in voided urine indicates the presence of tumor cells in the urinary tract. The results of the follow-up in time for our patient are indicated in Additional file 1. We demonstrate that the S249C mutation in *FGFR3 *detected in the tumor was also detected in the urine (Figure 1C), indicating that the detected tumor cells were shed by the resected tumor. Our results demonstrate that urine cytology does not detect the tumor in two cases, which is in concordance with previous studies that demonstrate a low sensitivity of urine cytology for the detection of tumors of low stage and grade [20,21]. Although this concerns just one patient, our results imply that patient monitoring by *FGFR3 *mutation analysis could be a feasible non-invasive method in the follow-up of young NMI-BC patients presenting with a mutation in *FGFR3 *and indicate that future research is required to investigate this.

## Conclusions

Multiple studies demonstrate the relatively benign disease course of bladder tumors in young patients, but there are some cases with a high recurrence rate and a progression rate comparable to older patients. We present a young patient having multiple multifocal recurrent bladder tumors with molecular characteristics found in older patients. It would be of interest to perform molecular studies in a larger subset of patients to elucidate whether these tumors comprise a biologically distinct group. Since BC tumors rarely present in young patients, a multicenter collaboration would be needed for investigation. This could ultimately lead to stratification of patients that need close monitoring and patients with favorable molecular characteristics of the tumor that can be monitored less frequently, hereby decreasing the number of cystoscopies performed in young patients who specifically perceive a cystoscopy as burdensome. For the follow-up of young patients presenting with an *FGFR3 *mutant NMI tumor, *FGFR3 *mutation analysis could be a feasible alternative for recurrence detection.

## Competing interests

The authors declare that they have no competing interests.

## Authors' contributions

TCMZ and ECZ designed the study. TCMZ performed the literature review and drafted the manuscript. CSA, MB and TCMZ collected the tissue and urine material. CSA, SST and KvdK performed *FGFR3 *mutation analysis and MA analysis. AvT performed the SNP-array and interpreted the results. MV performed immunohistochemical analysis and reviewed histopathological diagnosis. ECZ revised the manuscript for important intellectual content. All authors read and approved the manuscript.

## Consent

Written informed consent was obtained from the patient for publication of this case report and any accompanying images. A copy of the written consent is available for review by the Editor-in-Chief of this journal.

## Pre-publication history

The pre-publication history for this paper can be accessed here:

http://www.biomedcentral.com/1471-2490/10/5/prepub

## Supplementary Material

Additional file 1 **Patient follow-up schedule in time**. Patient's surveillance schedule starting at the primary tumor. Next rows represent tumor histology or urine cytology, *FGFR3 *mutation status and type of intravesical treatment. Click here for file
