# Supplementary material for: In-depth investigation of the molecular pathogenesis of bladder cancer in a unique 26-year old patient with extensive multifocal disease: a case report
Source: BMC Urol. 2010 Feb 26;10:5. doi: 10.1186/1471-2490-10-5 (PMC2843683; doi:10.1186/1471-2490-10-5)
Supplement: Supplementary file 1 [file 1471-2490-10-5-S1.DOC]

	T	U	T	U	T	U	T	U	U	U	T	U
**Sample date**	27-03-2007	12-06-2007	12-06-2007	02-10-2007	02-10-2007	28-11-2007	28-11-2007	28-03-2008	26-05-2008	11-11-2008	25-11-2008	14-01-2009
**Pathology/ Cytology**	TaG1	atypia	TaG2	no malignancy	TaG1	ND	TaG1	no malignancy	ND	no malignancy	TaG1	atypia
***FGFR3* status**	S249C	S249C	S249C	S249C	S249C	S249C	ND	negative	negative	S249C	ND	negative
**Intavesical therapy**			Epirubicin					BCG	BCG	MMC	Epirubicin	MMC

T Tumor
U Urine
ND Not Done
